# Comparison of bacterial species and clinical outcomes in patients with diabetic hand infection in tropical and nontropical regions

**DOI:** 10.21203/rs.3.rs-3831828/v1

**Published:** 2024-01-08

**Authors:** Yan Chen, Bin Liu, Chen Huan, Puguang Xie, Chenzhen Du, Shunli Rui, Mei Hao, Zixiao Duan, David G. Armstrong, Wuquan Deng, Xiaoqiu Xiao

**Affiliations:** the First Affiliated Hospital of Chongqing Medical University; Chongqing University Central Hospital; Bazhong city central hospital; Chongqing University Central Hospital; Chongqing University Central Hospital; Chongqing University Central Hospital; Renmin University of China; Chongqing University Central Hospital; Keck School of Medicine of University of Southern California; Chongqing University Central Hospital; the First Affiliated Hospital of Chongqing Medical University

**Keywords:** bacterial distribution, diabetic hand, tropical regions, nontropical regions

## Abstract

**Purpose:**

Hand infection is a rare complication in patients with diabetes. Its clinical outcomes depend on the severity of hand infection caused by bacteria, but the difference in bacterial species in the regional disparity is unknown. The purpose of this study was to explore the influence of tropical and nontropical regions on bacterial species and clinical outcomes for diabetic hand.

**Patients and Methods:**

A systematic literature review was conducted using PubMed, EMBASE, Web of Science, and Google Scholar. Moreover, the bacterial species and clinical outcomes were analyzed with respect to multicenter wound care in China (nontropical regions).

**Results:**

Both mixed bacteria (31.2% vs. 16.6%, p=0.014) and fungi (7.5% vs. 0.8%, p=0.017) in the nontropical region were significantly more prevalent than those in the tropical region. *Staphylococcus* and *Streptococcus* spp. were dominant in gram-positive bacteria, and *Klebsiella*, *Escherichia coli*, *Proteus* and *Pseudomonas* in gram-negative bacteria occupied the next majority in the two regions. The rate of surgical treatment in the patients was 31.2% in the nontropical region, which was significantly higher than the 11.4% in the tropical region (p=0.001). Although the overall mortality was not significantly different, there was a tendency to be increased in tropical regions (6.3%) compared with nontropical regions (0.9%). However, amputation (32.9% vs. 31.3%, p=0.762) and disability (6.3% vs. 12.2%, p=0.138) were not significantly differentbetween the two regions.

**Conclusion:**

Similar numbers of cases were reported, and the most common bacteria were similar in tropical and nontropical regions in patients with diabetic hand. There were more species of bacteria in the nontropical region, and their distribution was basically similar, except for fungi, which had differences between the two regions. The present study also showed that surgical treatment and mortality were inversely correlated because delays in debridement and surgery can deteriorate deep infections, eventually leading to amputation and even death.

## 1. INTRODUCTION

There are few peripheral artery diseases and neuropathic diseases involving the hand.^1^ Therefore, the incidence of diabetic hand disease is much lower than that of diabetic foot disease and is often ignored.^2,3^ Because of long-term hyperglycemia, impaired immune function, decreased neutrophil activity and abundant blood supply, hands or fingers are extremely susceptible to infection after injury.^4^ Even prompt treatment may lead to loss of function or amputation. Thus, diabetic hand disease should be well recognized by the public and health providers worldwide. Given its geographical localization and typical clinical features, diabetic hand infection used to be confined and reported in the tropics before. Gill et al. reported the disease known as tropical diabetic hand syndrome in 1998.^5^ These cases were diagnosed and reported mainly in African countries because of the hot climate,^3^ which is more suitable for the growth of bacteria. African women were predominantly manual workers, and hand infections were common after an injury.^5^ However, hand infections were not generally recognized as a specific diabetic problem in Western countries, as Dr. Gill described in 1998.^6^ In recent years, in both tropical and nontropical countries, diabetic hands have increased significantly. Thus, the definition of tropical diabetic hand syndrome is gradually being modified. The clinical outcomes depend on the severity of bacterial infection, geographical distribution and health service. Until now, there has been no comparison of bacterial species for diabetic hand in tropical and nontropical settings. In the present study, we investigated regional differences in the bacterial species of the diabetic hand. Furthermore, a multicenter study of bacterial species and clinical outcomes in patients with diabetic hands was also analyzed and reported in China.

## 2. PATIENTS AND METHODS

### 2.1 Literature review

The relationship between the bacterial diversity of diabetic hand infections and clinical outcomes is unknown through these case reports in tropical and nontropical regions. Therefore, we divided the tropical and nontropical groups according to regional division and literature sources (**Supplement 1**). We searched for articles between 1977 and 2022. The keywords used during the PubMed, EMBASE, Cochrane Library and Google Scholar searches were (hand) AND (diabetes OR diabetic) AND (infected OR infection OR ulcer).

### 2.2 Inclusion and exclusion criteria

Studies were included if they satisfied the following inclusion criteria: (1) study participants must have had a confirmed diagnosis of diabetic hand based on clinical symptoms, signs or imaging tests; (2) eligible patients had either a history of diabetes or a new diagnosis of diabetes; and (3) given that there are no RCTs on diabetic hand infection at present, the types of articles included could be case reports, observational studies, or retrospective studies.

**Figure 1:**
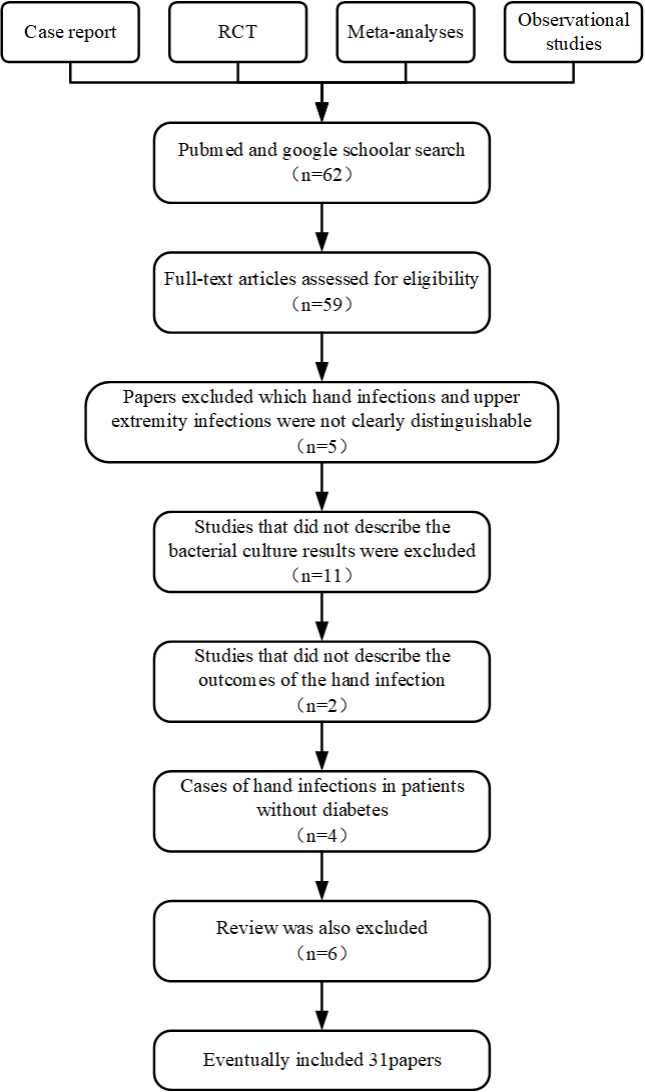
Flow chart of article screening for literature review

The exclusion criteria were as follows: (1) papers written in languages other than English; (2) hand infections and upper extremity infections were not distinguishable; (3) the results of bacterial culture or the outcomes of the hand infection were not described; (4) cases of hand infections in patients without diabetes; and (5) reviews were also excluded (Fig. 1). Two authors separately selected titles and abstracts and subsequently full-text articles. Disagreements were discussed with the third author, and inconsistencies were resolved after reaching a consensus.

### 2.3 Case collection in multicenter wound care

We retrospectively collected data from 14 patients with diabetic hand infection who were hospitalized in four hospitals in Chongqing, China, between May 2016 and May 2023. We summarized the basic medical history of these cases, time from onset to presentation, bacterial culture results, and final outcomes to explore the relationship between bacterial distribution and clinical outcomes in a nontropical region.

### 2.4 Statistical Analysis

SPSS version 22.0 (IBM SPSS Company, USA) was used for the statistical analysis. The nonnormal measurement data for multicenter wound care in China are expressed herein as the median (interquartile range) [M (P 25, P75)], and the Kruskal–Wallis H test was used to analyze the differences between the survival group and the nonsurvival group. The categorical data are expressed as percentages, and the differences between groups were analyzed using the chi square test and Fisher’s exact test. All statistical tests were two-sided, and p < 0.05 represented a significant difference.

## 3. RESULTS

### 3.1 General characteristics of the patients

In the study, a total of 31 publications^2,7–36^ were included in the analysis **(Supplement 2)**. A total of 713 patients with diabetic hand infections were included. The patients were divided into two groups as follows: a) Tropics Group, patients with diabetic hand infection in tropical regions (16 studies, n=367); b) Non-Tropics Group, patients with diabetic hand infection in nontropical regions (15 studies, n=346).

### 3.2 The difference in diabetic hand in nontropical and tropical regions

#### 3.2.1 .

There was no difference in etiologies between the two regions. The main causes included three categories: 1) trauma; 2) unknown causes or no history of injuries; and 3) postoperative or iatrogenic causes.^33–41^

#### 3.2.2 .

**Figure 2:**
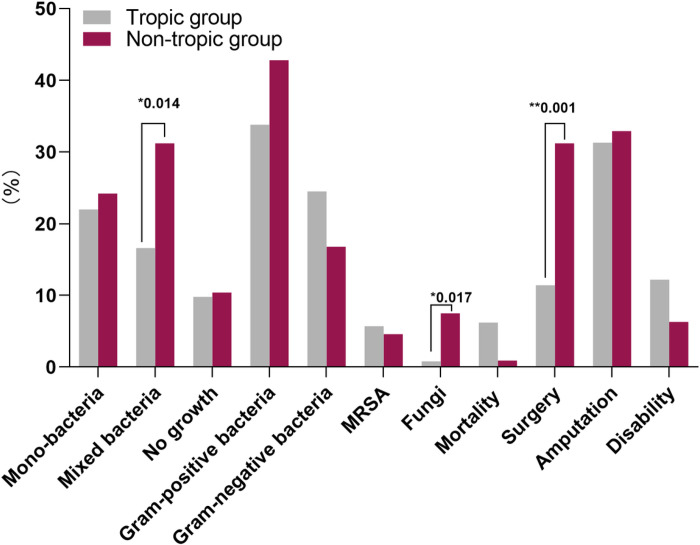
The characteristics of bacterial specie and outcomes of diabetic hand in tropical and nontropical group

According to the analysis of results reported in the literature (Table 1, Figure 2), both mixed bacteria (31.2% vs. 16.6%, p=0.014) and fungi (7.5% vs. 0.8%, p=0.017) in nontropical regions were significantly higher than those in tropical regions. However, the culture results of mono-bacterial growth (24.2% vs. 22%, p=0.73), MRSA (4.6% vs. 5.7%, p=0.756), and no bacterial growth (10.4% vs. 9.8%, p=1.0) were not significantly different between the two regions.

#### 3.2.3 .

Although the gram-positive bacteria were not significantly different, they were the most common bacterial isolates in nearly half of the cultures (33.8% in tropical regions and 42.8% in nontropical regions), which mainly included *Staphylococcus aureus* (Table 2), while gram-negative bacteria (24.5% vs. 16.8% p=0.165) in tropical regions and nontropical regions mainly included*Klebsiella, Pseudomonas, Escherichia coli* and *Proteus*.

#### 3.2.4 .

The difference in clinical outcome: The rate of surgical treatment in the patients was 11.4% in tropical regions, which was significantly lower than the 31.2% in nontropical regions (p=0.001). Based on the cases reported over the years, the overall mortality was 6.3% in tropical regions and 0.9% in nontropical regions, and there was a higher trend without a significant difference (p=0.054). However, amputation (31.3% vs. 32.9%, p=0.762) and disability (12.2% vs. 6.3%, p=0.138) were not significantly different between the two regions (Table 1, Figure 2).

#### 3.2.5 .

Clinical outcomes and bacterial species in a multicenter of wound care in a nontropical region: Fourteen patients with diabetic hand from four wound care centers in China were enrolled. The clinical outcome and bacterial species in China as a nontropical region are shown in **Table 3**. During the duration of hospitalization, 2 (14.3%) patients underwent amputation, while 4 (28.6%) patients passed away. Moreover, we compared the clinical characteristics between the survival and nonsurvival groups. The results indicated that there was no significant difference in age, diabetes duration, delayed admission, or HbA1c and CRP levels (all p>0.05).

### 3.3 A series of typical cases of diabetic hand are listed as follows:

#### Case 1:

**Figure 3:**
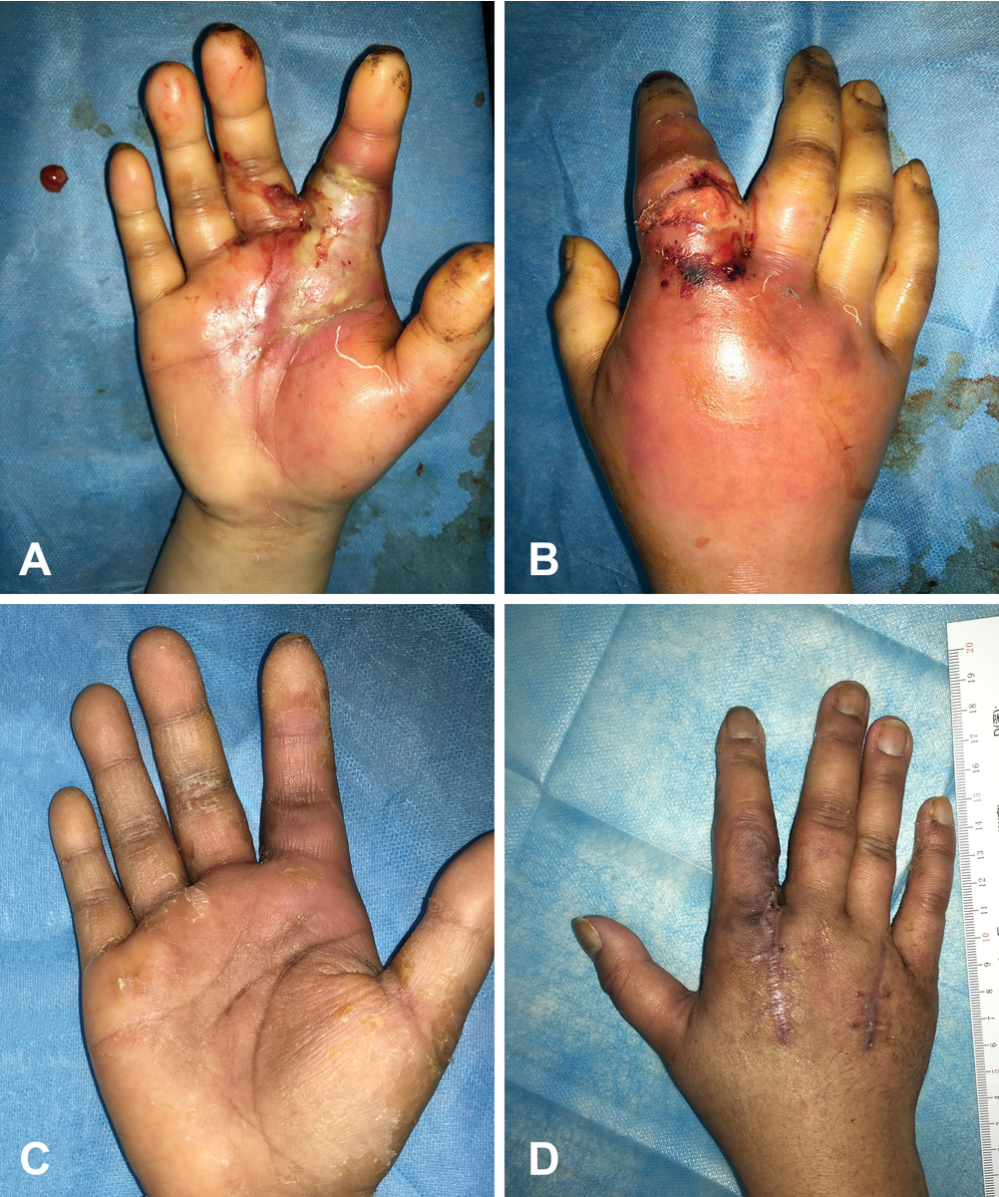
The patient recovered from severe hand infection with diabetic ketoacidosis after prompt treatment

A 27-year-old female was diagnosed with type 1 diabetes of 15 years’ duration with poor glucose control. She worked in a supermarket, and her right index finger was accidentally injured while she was moving items. After a simple disinfection treatment, the hand infection gradually deteriorated. The community physician treated the hand with a topical anti-infection cream one week later. Unfortunately, the condition of the infected hand was unmanageable, and the patient developed diabetic ketoacidosis one more week later. Therefore, she was transferred to our emergency room. Her right index finger showed swelling extending to the palm and dorsum of the right hand (Figure 3A–B). Her blood glucose, glycated hemoglobin (HbA1c) and β-hydroxybutyric acid levels reached 27 mmol/L, 17.2% and 4.8 mmol/L, respectively. She underwent aggressive surgical debridement with incisional drainage. The dorsal spaces were incised, exposed and allowed to drain freely. The culture of the debrided tissue revealed *Staphylococcus aureus*. After debridement and drainage, the wound was treated with vacuum-assisted closure four times, and we changed the dressing intermittently until she had healed. The patient was discharged from the hospital after 32 days. Her hand function and appearance were normal after treatment (Figure 3C–D).

#### Case 2:

**Figure 4:**
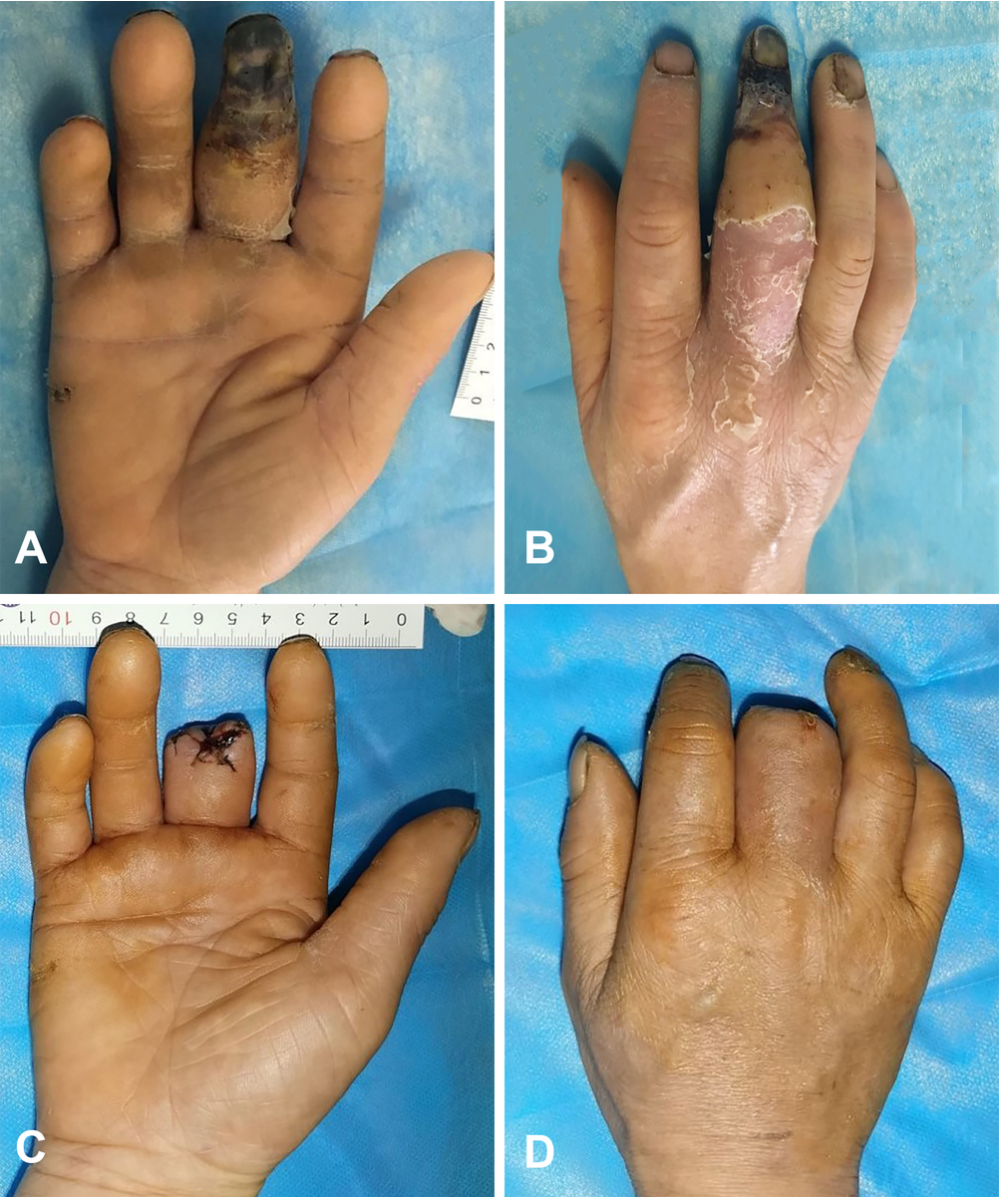
The diabetic hand suffered from amputation due to an improper treatment

A 43-year-old man with a history of uncontrolled diabetes mellitus presented to the emergency department with swelling, darkened skin and a darkened nail in the right hand. One month prior, his right middle finger was accidentally stabbed by a barbecue bamboo skewer. The finger gradually became red, swollen and numb and finally underwent necrosis after being soaked in a mixed solution of salt, vinegar and garlic for 4 hours at home. Then, the finger was treated by acupuncture and blood-letting therapy with a needle in a private clinic. During this period, the patient continued to soak his hand with the above mixed solution. His fingertip condition progressed to tissue necrosis and gangrene with a foul smell. Physical examination revealed spindle swelling at the proximal phalanx of the middle finger, and the fingertip showed total dry gangrene and many needle holes (Figure 4A–B). Radiographs showed slight osteomyelitis at the proximal phalanx of the finger. Amputation was performed at the distal phalanx of the finger after normalization of his blood glucose. The culture of bone tissue at the surgical site was positive for *Morganella morganii* and *Proteus penaeus*. Postoperatively, the patient received intravenous clindamycin for two weeks. The patient’s right hand was disabled after the amputation surgery (Figure 4C–D) and after he was discharged from the hospital.

#### Case 3:

**Figure 5:**
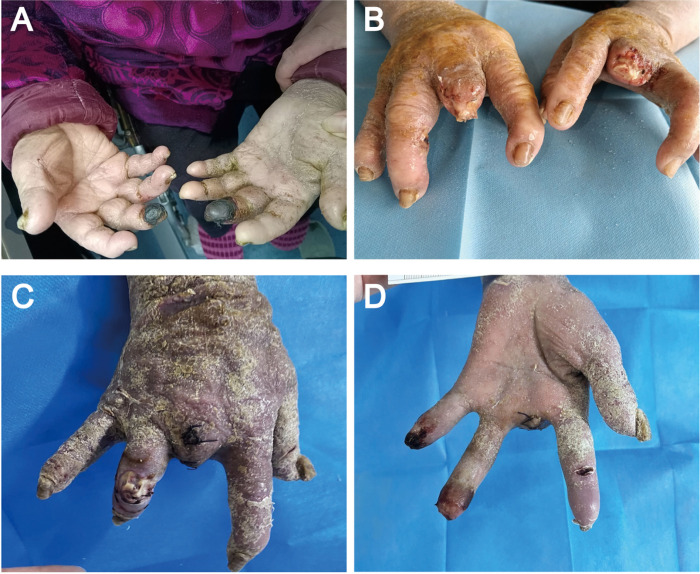
The diabetic patient with chronic renal failure died by gastrointestinal hemorrhage after hand infection

A 71-year-old woman presented with a 21-year history of diabetes and eight years of hemodialysis because of kidney failure due to diabetic nephropathy. Both of her hands were initially diagnosed with distal middle finger gangrene (Figure 5A). The proximal finger was infected after surgery to remove the distal gangrene, and there was a purulent discharge (Figure 5B). The hand infection worsened gradually until the entire middle finger was removed (Figure 5C–D). A bacterial culture of the discharge revealedMRSA infection. The patient was treated with wound debridement and dressing changes. At the same time, she was treated with insulin, antihypertensive drugs, antibiotics and continuous hemodialysis three times weekly. Her other fingers also began to develop ulcers gradually. However, she was discharged 2 months later against the doctor’s advice. Unfortunately, a recent follow-up revealed that she died due to gastrointestinal hemorrhage resulting from chronic renal failure.

## 4. DISCUSSION

Previously, in Africa, the mortality from diabetic hand infection was very high. All four patients reported in Tanzania died in 1997.^42^ Due to the poverty status and low education level of patients in tropical countries, they were not aware of the dangerousness and seriousness of diabetic hand. A delay in seeking medical advice or in being referred to the hospital as well as a lack of hand care usually occur worldwide. As one of the diabetic patients described in Case 2, he dealt with his infected finger by using an unscientific approach at home; unfortunately, his finger developed gangrene that necessitated an amputation after his delayed admission. In addition, the disease, usually handled without timely and appropriate medical treatment methods, is poorly understood by clinicians. ^43^ In Case 1, we described a patient with diabetes whose hand was infected because of her poor control of blood glucose. The local community physician only gave her a prescription for a topical anti-infection cream. After one week of treatment, the patient was almost in a coma and presented to the emergency department. Then, she was administered insulin therapy for DKA. Furthermore, her finger was urgently debrided thoroughly. Therefore, identification of the etiology and monitoring of bacterial species followed by targeted use of antibiotics are very important for diabetic patients with hand infections.

In contrast to a previous report,^1^ we found that *Staphylococcus aureus* was predominant in both regions. *Streptococcus* is the second-most prevalent bacteria in nontropical regions and comprises a large group of pyogenic gram-positive bacteria with high pathogenicity. Therefore, 31.2% of patients with diabetic hand infections have to undergo surgical treatment in nontropical regions, which mostly includes thorough debridement, incisional drainage and even finger amputation when they are admitted to the hospital. A comprehensive treatment strategy has led to a better prognosis and lower mortality ^42^ in nontropical regions. Of course, the education level of patients and medical technology of the hospital might have played important roles in the prevention and treatment of diabetic hand infection.^44^

The prevalence of mixed bacteria (31.2%) and fungi (7.5%) was significantly higher in nontropical regions than in tropical regions. Some studies reported that multiple bacterial infections were more serious, with a longer hospital stay and more surgeries with a higher rate of amputation. ^1,45–47^ A similar finding was observed in our study. Interestingly, the mortality is lower in the nontropical regions. This could be explained by early and aggressive treatment for reducing mortality, even if the involved bacteria are more complex.^46^ Meanwhile, accurate sampling methods are vital. Sangeeta Tiwari reported^3^ that cultures of tissue biopsy specimens produced a single bacterial species in 75% of cases, whereas swab cultures produced mixed bacteria in most cases, which may be cultured as a result of contamination.^48^

MRSA is a kind of virulent bacteria, and it has even been called a superbug.^49^ The treatment of MRSA infection is one of the most difficult issues in the clinic due to its multidrug resistance to most antibiotics. Because there are more MRSA infections and worse medical care in some developing countries of the tropical region, this may be one reason for the higher mortality. The two patients at our center who suffered from MRSA also had outcomes of amputation and death.

Diabetic hand infections in tropical regions have been reported to involve 14 kinds of bacteria compared to 22 in nontropical regions. There were more species of bacteria in nontropical areas, which may be because some of the articles from the tropical areas did not specify the bacterial isolates in detail within the study^40,50^ or did not list the bacterial culture results at all ^42^. In addition, this difference may have been caused by different climates and environments.

The incidence of diabetic hand infections is lower than the approximately 50–60% of diabetic foot ulcers (DFUs)^51^. The most important factor in the pathogenesis of DFU is also infection ^52^. There are also regional differences in bacterial species and clinical outcomes. The most common bacterial isolates in DFUs are aerobic gram-positive cocci, especially *S aureus*, but DFUs in patients from tropical climates often have aerobic gram-negative bacilli isolated^53^.

Except for the type of bacterial isolates, the factors affecting clinical outcome mainly involved the duration of diabetes, the glycated hemoglobin level,^39–40^ end-stage renal disease (diabetic hand infection patients who were kidney recipients all underwent amputation^33^), sepsis, the depth of the infection (necrotizing fasciitis had a worse prognosis than a superficial infection), and smoking.^40^ Medical technology and precise usage of systemic antibiotics are also crucial for prognosis;^11^ thus, there were many contributing factors affecting the outcome, other than the species of bacteria. It has been reported that the factors mentioned above are more important than climate or geography, which leads to poor prognostic outcomes^44^.

Although other studies documented that there was no significant association between death and delay in presentation^45^, our experience and numerous previous reports showed that early intervention and timely and effective debridement are crucial for diabetic hand infection. ^11,46^ The surgical case percentage and mortality are in the reverse direction, which proves this conclusion since a delay in debridement and surgery can deteriorate deep infections, which in turn may eventually lead to amputation and even death.^46^ Notably, the few deaths we reported were due to other serious comorbidities, such as malignancy, renal failure, and gastrointestinal bleeding, rather than to the hand infection itself. Most cases in our center were treated with systemic antibiotics, correction of metabolic disorders, aggressive debridement and drainage, and timely control of the infection.

## 5. CONCLUSION

The prevalence and number of patients reported were similar in tropical and nontropical regions.

There was no significant difference in the etiology between the two regions, as we found that *Staphylococcus aureus* dominated in both regions. Both mixed bacteria and fungi in nontropical regions were significantly more prevalent than those in tropical regions. Surgical treatment is more commonly used in nontropical regions, and therefore, the overall mortality in nontropical regions had a downward tendency. However, due to the varying severity of diseases, there are significant differences in treatment time and methods among different studies, and some regions have insufficient medical experience. Consequently, these conclusions still need to be validated through large-scale, high-quality randomized controlled trials.

## Figures and Tables

**Table 1.** The characteristics of bacterial specie and outcomes in patients with diabetic hand in tropical and nontropical group

Characteristics	Tropics group (n=367)	Non-tropics (n=346)	*p*-value
Paper number	16	15	/
Mono-bacteria n (%)	81 (22%)	84 (24.2%)	0.737
Mixed bacteria n (%)	61 (16.6%)	108 (31.2%)	0.014
No growth n (%)	36 (9.8%)	36 (10.4%)	1
Gram-positive bacteria n (%)	124 (33.8%)	148 (42.8%)	0.191
Gram-negative bacteria n (%)	90 (24.5%)	58 (16.8%)	0.165
MRSA n (%)	21 (5.7%)	16 (4.6%)	0.756
Fungi n (%)	3 (0.8%)	26 (7.5%)	0.017
Mortality n (%)	23 (6.3%)	3 (0.9%)	0.054
Surgery n (%)	42 (11.4%)	108 (31.2%)	0.001
Amputation n (%)	115 (31.3%)	114 (32.9%)	0.762
Disability n (%)	45 (12.2%)	22 (6.3%)	0.138

**Table2.** The bacterial species in patients with diabetic hand in tropical and nontropical regions

Climatic Regions	Organisms	Patients

**Tropic**	Staphylococcus aureus	86

	Klebsiella	39

	Streptococcus	36

	Escherichia coli	10

	Pseudomonas	9

	Proteus	9

	Enterobacter	12

	Nonformative GNB	5

	Acinetobacter	2

	Citrobacter diversus	2

	Fungi	3

	Enterococcus	2

	Bacteroides fragilis	1

	Aeromonas	1

**Non-tropic**	Staphylococcus aureus	100

	Streptococcus spp	30

	Fungi	26

	Klebsiella	11

	Proteus	8

	Enterobacter	8

	Pseudomonas	7

	Enterococcus	7

	Escherichia coli	5

	Diphtheroids	5

	Mycobacterium	4

	Serratia	3

	Pasteurella multicoda	3

	Citrobacter	3

	Clostridium Perfingens	2

	Morganella morganii	2

	Bacteroids	2

	Acinetobacter	2

	Vibrio vulnificus	1

	Micrococcus	1

	Eikenella	1

	Corynebacterium	1
	

## Data Availability

The datasets generated during this study are available from the corresponding author upon reasonable request.

## Key messages

1. The differences in bacterial species and clinical outcomes among tropical and nontropical regions are still unclear.
2. Both mixed bacteria and fungi in nontropical regions were significantly more prevalent than those in tropical regions.
3. Surgical treatment was more commonly used in nontropical regions, so the overall mortality in nontropical regions had a downward tendency.
